# Metastatic Crohn Disease Manifesting as Granulomatous Cheilitis in a Patient With Well-Controlled Crohn Disease

**DOI:** 10.14309/crj.0000000000002135

**Published:** 2026-05-22

**Authors:** Hamza Almusabeh, Katherine Shepherd, Khalid Elfert, Rehmat Awan, Colleen Beatty, Kanwarpreet Tandon, Jennifer Hadam-Veverka

**Affiliations:** 1 Department of Internal Medicine, West Virginia University School of Medicine, Morgantown, WV; 2 Division of Gastroenterology and Hepatology, West Virginia University School of Medicine, Morgantown, WV; 3 Department of Dermatology, West Virginia University School of Medicine, Morgantown, WV

**Keywords:** Crohn's disease, metastatic Crohn's disease, inflammatory bowel disease, orofacial Crohn's disease

## Abstract

Crohn disease (CD) can affect any organ system outside the gastrointestinal tract, and these conditions are classified as extraintestinal manifestations and include arthritis, uveitis, and cutaneous manifestations. Cutaneous involvement includes erythema nodosum, pyoderma gangrenosum, and metastatic CD. The latter can be defined as a granulomatous skin lesion that is noncontiguous to the intestinal disease. In this article, we report a rare case of a 48-year-old woman with CD in endoscopic and clinical remission with chronic persistent orofacial granulomatosis of the lip secondary to CD, despite the absence of active intestinal disease and describe its management.

## INTRODUCTION

Crohn disease (CD) is a chronic inflammatory granulomatous disorder primarily affecting the gastrointestinal tract and is usually characterized by a discontinuous pattern of lesions. CD can affect any organ system outside of the gastrointestinal tract. These manifestations include arthritis, uveitis, and cutaneous manifestations.^1^ Cutaneous involvement includes erythema nodosum, pyoderma gangrenosum, and metastatic CD (MCD).^2^ The latter is defined as a granulomatous skin lesion noncontiguous with the intestinal disease.^2,3^ In the literature, orofacial CD (OFC) is described as an exceedingly rare entity of MCD, with fewer than 10 cases reported.^4^ We report a case of CD with active cutaneous granulomatous cheilitis in the absence of active intestinal disease and describe its management.

## CASE REPORT

**Figure 1.**
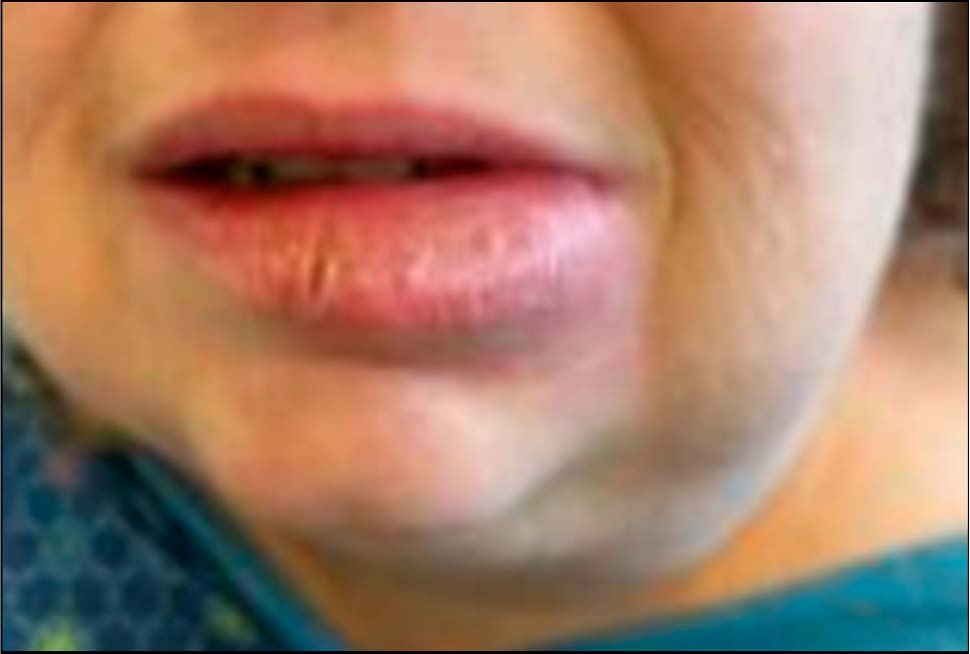
Lateral erythema, vertical furrowing, and swelling of the lower lip.

**Figure 2.**
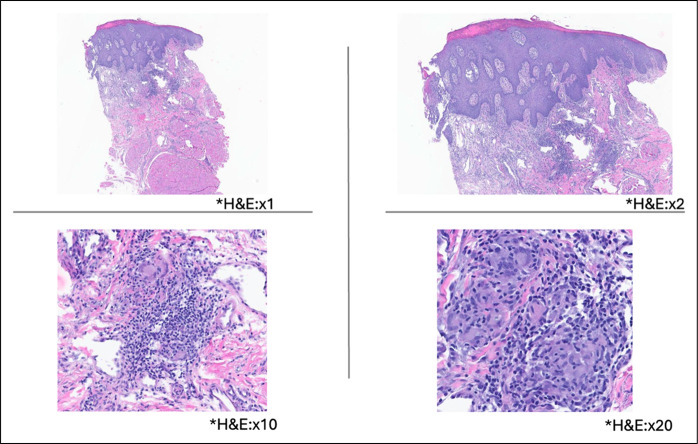
H&E of punch biopsy from the lip showed psoriasiform hyperplasia overlying small collections of histiocytes and multinucleated epithelioid giant cells within the mid dermis. These granulomas are surrounded by a moderately dense mixed inflammatory infiltrate. *Photomicrographs magnification. H&E, hematoxylin and eosin.

A 48-year-old woman with a medical history significant for asthma, fistulizing ileocolonic CD diagnosed at age 10 years, who later required a proctocolectomy with end-ileostomy for medically refractory disease. She has been maintained on adalimumab (ADA) 40 mg every 7 days since 2014, which led to the successful closure of her fistula without evidence of active luminal disease since 2019. The most recent inflammatory markers from 2025 included a C-reactive protein of 9 (normal ≤ 8) and fecal calprotectin (181 mcg/g). Her last ileoscopy in 2019 and computed tomography (CT) enterography in 2023 were both normal. In addition, ADA levels were 10.8 μg/mL, and ADA antibodies were undetectable. Despite controlled intestinal disease, the patient reported recurrent lip swelling and itching since 2018, which had not previously been addressed. The dermatologist's evaluation revealed lateral erythema, scaling, and swelling of the lips with vertical furrows (Figure 1). Initially, no biopsy was obtained, and her lip lesion was treated empirically with topical desonide 2 times daily as needed. Owing to persistent symptoms, a biopsy of the lip lesion was obtained, which showed dermal granulomatosis with overlying psoriasiform hyperplasia, small collections of histiocytes, and multinucleated epithelioid giant cells within the mid-dermis. These granulomas are surrounded by a moderately dense mixed inflammatory infiltrate composed of lymphocytes, mast cells, plasma cells, and scattered eosinophils. In addition, a periodic acid-Schiff stain was negative for fungal infection (Figure 2). The clinical picture, along with the histological findings, supports the diagnosis of granulomatous cheilitis in CD. She was prescribed topical tacrolimus in addition to 0.05% desonide cream and ADA. At her 6-month follow-up, her swelling had significantly improved, but it was not completely resolved.

## DISCUSSION

MCD is considered an uncommon skin manifestation of CD, with OFC involvement among the rarest locations of MCD.^4,5^ Granulomatous cheilitis has rarely been reported in association with CD in adult patients.^6,7^ Our case is notable because it occurred in the setting of clinically and endoscopically quiescent intestinal disease despite therapeutic ADA levels and the absence of antidrug antibodies.^8^ The diagnosis can be challenging as OFC can manifest independently of gastrointestinal symptoms and disease activity. Approximately 20% of patients with MCD have no history of gastrointestinal symptoms. A biopsy demonstrating a noncaseating granuloma is required to confirm the diagnosis.^2^ When considering the clinical and histological findings in our patient, the most likely diagnosis is orofacial granulomatosis secondary to CD. Although sarcoidosis can have a similar presentation, the diagnosis is unlikely in our patient because of the absence of hilar lymphadenopathy and lung fibrosis in her most recent CT of the chest. In addition, calcium levels were within normal limits.^9^ Other diagnostic considerations included Melkersson-Rosenthal syndrome, idiopathic orofacial granulomatosis, an infectious cause, and a foreign body reaction, although these were considered less likely given the patient's established history of CD, compatible histological findings, and a negative periodic acid-Schiff stain.

Cutaneous lesions of MCD have been reported in different anatomical locations, including the lips, penile, labial, skin folds, or multiple sites concurrently.^2,10–13^ Based on a systematic review of 264 biopsy-proven MCD cases, the genitalia are the most common location of the disease. The duration of MCD lesions has been reported to last up to 21 months with treatment.^13^

The management of MCD is highly challenging as it can manifest despite well-controlled intestinal disease. Our patient was in clinical and endoscopic remission with ADA levels in the therapeutic range without antibody formation, and despite that, she had the cutaneous manifestations. A change in biologic therapy was not initially pursued because the fecal calprotectin elevation was not considered sufficient to justify a change in therapy in the absence of other evidence of active intestinal disease. Furthermore, MCD may persist for a prolonged period before showing improvement.

Given the rarity of MCD and the scarcity of high-quality studies on its management, multidisciplinary management, including dermatology, is highly recommended.

Currently, the management is based on low-quality evidence, including case reports and series. Studies have reported partial improvement with some monotherapies, including anti-TNF alpha, thiopurines, systemic corticosteroids, and metronidazole. On the other hand, other studies suggest combination therapy, including metronidazole with systemic corticosteroids or tacrolimus, as well as surgical intervention.^13^ Our patient was initially started on topical steroid monotherapy with no significant improvement. However, partial resolution of her lips was achieved after she was started on combination therapy with topical steroids and tacrolimus. If the patient does not demonstrate further clinical improvement, future therapeutic considerations may include transition to an agent with a different mechanism of action, such as an IL-23 inhibitor, a Janus kinase inhibitor, or an IL-12/23 inhibitor. Capsule endoscopy may also be considered if symptoms persist, particularly if there is concern for occult small-bowel disease not detected in prior evaluation; however, this was not pursued initially, given the absence of gastrointestinal symptoms and otherwise reassuring ileoscopy and CT enterography findings.

This case report highlights an atypical location of MCD, which itself is a rare cutaneous manifestation of CD. We emphasize that providers should maintain a high index of suspicion for MCD in patients with persistent cutaneous lesions, even in the presence of both clinical and endoscopic remission of intestinal manifestations. In addition, this report suggests that combination therapy may be more effective than monotherapy in treating OFC involving the lips.

## DISCLOSURES

Author contributions: H. Almusabeh and K. Shepherd, involved in the literature review and drafting the manuscript. K. Elfert and R. Awan assisted with endoscopic image acquisition, interpretation, and the review of the draft manuscript. C. Beatty assisted with interpreting pathological images and writing the description. K. Tandon and J. Hadam-Veverka helped in the critical appraisal of the manuscript and is the article guarantor.

Financial disclosure: None to report.

Informed consent was obtained for this case report.

## ABBREVIATIONS:

ADA: adalimumab
CD: Crohn's disease
CT: computed tomography
OFC: orofacial CD
